# Genetic Approaches for Neural Circuits Dissection in Non-human Primates

**DOI:** 10.1007/s12264-023-01067-0

**Published:** 2023-06-01

**Authors:** Ling Li, Zhen Liu

**Affiliations:** 1 Institute of Neuroscience, CAS Center for Excellence in Brain Science and Intelligence Technology, CAS Key Laboratory of Primate Neurobiology, State Key Laboratory of Neuroscience, Chinese Academy of Sciences, Shanghai, 200031 China; 2 Shanghai Center for Brain Science and Brain-Inspired Intelligence Technology, Shanghai, 200031 China; 3 University of Chinese Academy of Sciences, Beijing, 100049 China

**Keywords:** Genetic tool, Neural circuit, Non-human primate

## Abstract

Genetic tools, which can be used for the morphology study of specific neurons, pathway-selective connectome mapping, neuronal activity monitoring, and manipulation with a spatiotemporal resolution, have been widely applied to the understanding of complex neural circuit formation, interactions, and functions in rodents. Recently, similar genetic approaches have been tried in non-human primates (NHPs) in neuroscience studies for dissecting the neural circuits involved in sophisticated behaviors and clinical brain disorders, although they are still very preliminary. In this review, we introduce the progress made in the development and application of genetic tools for brain studies on NHPs. We also discuss the advantages and limitations of each approach and provide a perspective for using genetic tools to study the neural circuits of NHPs.

## Introduction

Thanks to recent advancements in molecular biology, cell biology, synthetic biology, gene editing, and virus studies, several powerful genetic tools have been developed for specific neuron labeling, circuit tracing, neuronal activity recording, and perturbing with high spatiotemporal resolution. The development of gene-editing methods makes it possible and efficient to generate sizable transgenic animal lines that target specific cell types. Various viral vectors with unique tropisms play crucial roles in delivering genetic material to different cell types and mediating anterograde/retrograde transport among neurons. Engineered opsins and designer receptors exclusively activated by designer drugs (DREADDs) enable the reversible control of activity in specific neuronal populations. Calcium indicators, equipped with microscopes, enable long-term and large-scale tracking of the activities of distinct cell types. The widespread application of these genetic tools in small animals such as rodents, zebrafish, and *Drosophila*, has allowed the precise dissection of neural circuits and has largely revolutionized the research paradigm in neuroscience.

Non-human primates (NHPs) are important animal models in neuroscience research, as they have brain network organization, cognitive circuits, brain structures (e.g., enlargement of the prefrontal cortex), and a proportion of neocortex similar to humans [1, 2]. In addition, NHPs are more similar to humans at cellular-molecular levels than rodents. For instance, a population of slow-spiking neurons expressing nitric oxide synthase (nNOS), neuropeptide Y (NPY), and GABA_A_ receptor α1 subunit (GABA_A_R-α1) -"ivy cells" - are abundant in the neocortex of primates, while in mice they are predominantly located in the hippocampus [3, 4]. The structural basis for supporting such a considerable operating system is the complex brain that consists of billions of neurons, trillions of synaptic connections, and specialized neural circuits. Efforts in unraveling the neural mechanisms of brain physiology and pathology in NHPs could deepen our understanding of the human brain. However, brain studies on NHPs are still limited. Traditional electrophysiology, pharmacological intervention, functional magnetic resonance imaging (fMRI), and intrinsic imaging have been widely used in NHPs to reveal the correlations and causal relationships between specific behaviors and neuronal activity. Yet, traditional methods lack the ability to precisely identify cells and are limited in how they can modulate and record cells. Genetic tools, as more powerful neuroscience approaches, have been lagging in NHPs due to various limitations. Here, we review the genetic tools and related advances that have been applied to primates, including cell-type targeting approaches, viral vector delivery technology, and genetic tools for neuronal activity manipulation and tracking. We believe that genetic approaches have the potential to open new horizons and to reveal the wiring logic and inner mechanisms of neural circuits involved in complex physiological and pathological functions.

## Cell-Type Specific Targeting in Monkey

**Fig. 1:**
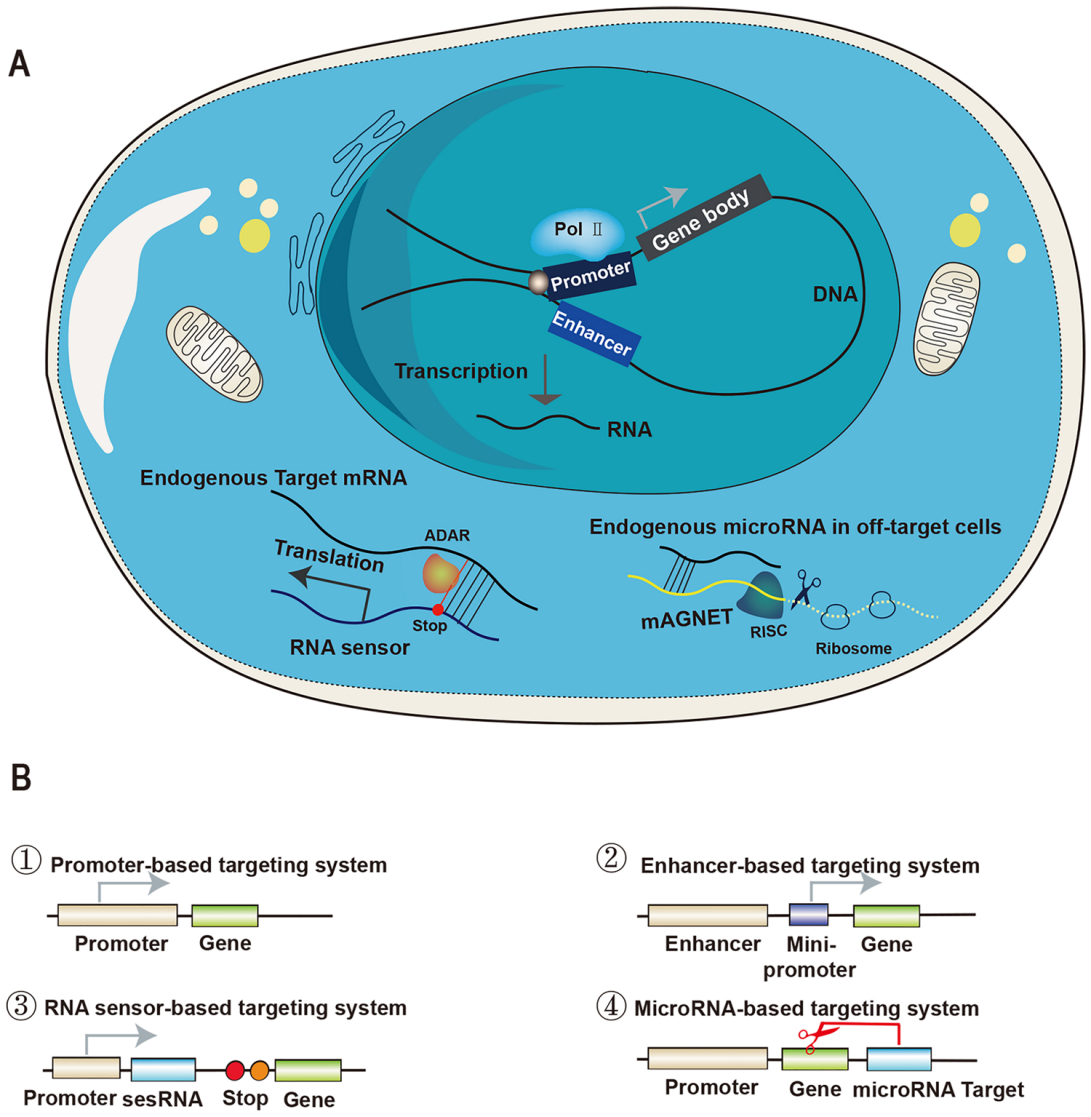
Genetic cell-type-targeting strategies *via* viral vectors. **A** Principle overview of several genetic strategies to target cell types. Promoter/enhancer-based approaches work at the gene transcription stage. Generally, the promoter (dark blue box) determines the transcription initiation sites, while the enhancer (sapphire blue box) determines the transcription efficiency of genes. Enhancers activated by specific TFs move close to their target promoter through a looping mechanism, and their interaction is stabilized by cohesion proteins (gray balls). RNA sensor/mAGNET work at the gene translation stage. In programmable RNA sensor tools, sesRNA hybridizes to target endogenous mRNA, and ADAR enzymes (orange ball) repair mismatched sites of the stop codon (red circle) switching on the translation of required proteins. For mAGNET, abundant exogenous miRNAs recruit the RISC (dark green sphere) and inhibit nonspecific protein translation by hybridizing to their complementary miRNA in off-target cells. **B** Sequence elements of four targeting approaches. Red scissors, repair sites; red circle, stop codon; orange circle, self-cleaving peptide T2A; TFs, transcription factors; Pol II, RNA polymerase II; mAGNET, miRNA-guided neuron tag; RISC, RNA silencing complex.

A fundamental technical problem in neuroscience studies is the fact that the nervous system is highly heterogeneous, consisting of many intermixed cells. Distinct cell types cannot be identified by a single criterion. Common classification criteria are molecular, morphological, and neurophysiological properties. The neural underpinnings of behaviors, including the information processing of parallel, convergent, divergent, feedback, and streamlining, are maintained by the assembly and the interactions of distinct cell types in the mammalian brain. Gaining access to different cell types is necessary in order to better understand how the brain works in NHPs. Recently, the rapid development of sequencing technology has provided scientists with a resource to finely identify cell types and gene regulatory networks. Fueled by innovations in genome editing techniques, genetic tools have a great inherent potential to distinguish and study specific cell types. Here, we discuss cell-type-targeting methods in primates and look ahead to new approaches to gain insight into the complexity of mesoscopic neural circuits in primates (Fig. 1).

### Cre Knock-in-Based Cell-Type Targeting

Conventional Cre knock-in lines, generated from embryonic stem cells by gene targeting, make it possible to target specific cell types reliably, revolutionizing the study of neural circuits in mice [5, 6]. The development of genome editing methods (zinc-finger nucleases, transcription activator-like effector proteins, and the CRISPR/Cas9 system) allows the generation of gene-modified monkeys [7, 8]. Gene-modified monkey disease models carrying indels or point mutations by CRISPR/Cas9 or a CRISPR-based base editor have been reported in many studies with high efficiency [9, 10]. Yet, the efficiency of precise large-fragment knock-in *via* homologous recombination in embryo gene editing is still relatively low, making it acceptable to generate knock-in mouse models, but not knock-in monkey models. To date, no Cre knock-in monkey model has been reported due to the low efficiency, high cost, and limited available resources. Alternative approaches, such as exogenous gene expression *via* local injection of viral vectors driven by promoters, enhancers, and other regulators have been so far considered more feasible. We recommend that efforts be made towards improving the efficiency of this approach and developing a long-term plan for the generation of Cre knock-in monkey models.

### Promoter-Based Cell-Type Targeting

A promoter is a DNA sequence upstream of a gene where relevant proteins, such as RNA polymerase II (RNAPII) and general transcription factors bind to the transcription start site of that gene. The function of the promoter is generally thought to be to determine the initial transcription location and direction of a single RNA transcript. The endogenous expression pattern of a gene of interest can be mimicked by inserting exogenous genes after a known promoter of this gene. Although the expression patterns of exogenous genes are far from satisfactory *in vivo*, the promoter-driven cell-type-targeting approach remains the simplest and quickest way to target specific neurons in monkeys today.

The promoter most widely used in monkeys is the Ca^2+^/calmodulin-dependent protein kinase II alpha (CaMKIIα). This gene is mainly expressed in excitatory neurons and its promoter preferentially targets the same cell type, enabling high-level transgene expression in excitatory neurons, especially in layer 5 of the neocortex [11, 12]. The human synapsin-1 promoter (hSyn) has an expression pattern similar to the CaMKIIα promoter in monkeys, resulting in particularly robust expression in the deep layers [11–14]. Other studies have also selected fused cytomegalovirus (CMV), human thymocyte-1(hThy-1), or the CMV enhancer, chicken β-actin promoter, and rabbit β-globin splice acceptor site (CAG) as the ubiquitous promoter [14]. In addition to the general cell type, the tyrosine hydroxylase (TH) promoter, coupled with the Cre/loxP system can be used to target TH^+^ neurons in the locus coeruleus and substantia nigra of primates [15]. The L7/Pcp2 promoter can be used to track Purkinje cells in the cerebellum of monkeys [16]. This strategy depends on the specificity of a native promoter. However, the expression pattern of most genes is not promoter dependent, leading to unspecific labeling in the promoter-driven cell-targeting approach. Indeed, the specificity of many promoters is likely the result of random integration next to highly specific *cis*-regulatory elements [17]. In conclusion, the promoter-driven cell-type-targeting approach can achieve some degree of specificity, but such specificity is relatively modest and largely falls short in the finer targeting of specific cell types.

### Enhancer-Based Cell-Type Targeting

Investigations of eukaryotic transcriptional regulation have revealed that the same gene can be expressed in distinct cell types *via* the activation and inactivation of different sets and combinations of enhancers or other *cis-*regulatory elements [12, 13]. Enhancers, typical genomic *cis*-regulatory elements, can modulate gene expression patterns in different tissues irrespective of their orientation or distance by a looping mechanism [18]. Recent progress in profiling the landscape of accessible chromatin of single cells has dramatically expanded our ability to identify enhancers [19]. Enhancer-driven gene expression (EDGE) can mimic endogenous marker gene expression patterns and transfer artificial genes into specific cell types by hijacking enhancers. Several studies have shown that EDGE can be used to generate useful genetic tools for accessing specific cell subclasses in rodents (e.g., layer 5 pyramidal tract neurons) [17, 20-25]. There are also a few reports on monkeys. A collection of parvalbumin (Pvalb) enhancers showing highly specific access to Pvalb cells in the primate neocortex has been defined by Mich *et al.* [26]. The mouse *Dlx5/6* enhancer has provided a powerful tool for GABAergic neuron manipulation in macaques [27]. An intersectional strategy using the human *Dlx5/6* enhancer and the somatostatin (Sst) promoter has confined the expression of exogenous proteins to Sst^+^ interneurons in the marmoset cortex and the rhesus macaque cortex [28]. Currently, the enhancer-based cell-type targeting system is the most powerful and simple genetic tool available to target specific cell types in primates. Future research towards building up a comprehensive toolkit of enhancers for precise cell-type labeling in monkeys would add another layer of sophistication to basic research on the NHP brain, and truly deepen our understanding of the assembly and organization of neural circuits and their operating principles.

### Programmable RNA-based Cell-Type Targeting

Engineered RNA-specific sensors have recently been shown to be promising for directly targeting cell types at endogenous mRNA levels. Several laboratories have reported independently programmable RNA sensing systems that harness endogenous adenosine deaminases acting on RNA (ADAR) repair systems to detect intracellular transcripts and target specific cell types [29-32]. In these systems, a programmable RNA sensor encodes the exogenous protein for which transcription is interrupted by artificially inserted stop codons in the context of a sequence complementary to the target transcript of the genes of interest. Only in the presence of target transcripts in a certain type of neuron, the sense–edit–switch RNA (sesRNA) would hybridize with the target mRNA and form double-stranded RNA molecules (dsRNA). The ADAR enzyme is then recruited to convert A to I at the mismatch sequence, switching on the expression of reporter proteins. The capacity of sesRNA in operating and tracking the activities of specific cell types has been proven in cultured cell lines, behaving mice, and human brain slices. Such a system, which bypasses complex and indirect DNA transcription processes, provides a relatively flexible and straightforward approach to target cell subclasses across species, especially for monkeys and humans [33].

### MicroRNA-Based Cell-Type targeting

The microRNA (miRNA)-based genetic system can also operate at endogenous mRNA levels. This approach – miRNA-guided neuron tag (mAGNET) – suppresses transgene expression in off-target cell populations to enable gene expression in specific cell types. Specific miRNA from off-target cells or tissues recruits the RNA-silencing complex (RISC) and degrades the transcripts by hybridizing to their complementary miRNA recognition sites incorporated in viral vectors. Conversely, low levels of miRNA in on-target cells have little impact on the translation of exogenous proteins. This strategy has been successfully applied to identify and label interneurons in rodents [34]. In addition, the miRNA-mediated de-targeting system is also used to down-regulate the transcripts of exogenous delivery proteins in the dorsal root ganglion (DRG) of primates with the aim of reducing toxicity and the immune response of AAV-mediated gene therapies [35]. Although not commonly used, the miRNA-mediated de-targeting approach offers another option for researchers to label general cell populations.

In summary, targeting specific cell types in NHPs remains a daunting challenge. Viral-mediated cell targeting methods can overcome the defects of traditional Cre knock-in monkey models in terms of time, cost, and resource requirements. Enhancers often outperform native promoters in their ability to target cell types with high stability and specificity. While EDGE has so far withstood many tests and is promising for cell-type targeting in NHPs, researchers often run into the issue of enhancer validation and elusive transcription regulation *in vivo*. Recently, engineered RNA sensors, a novel genetic tool, have made tag events occur during the mRNA-based translation process. This system is convenient and flexible in terms of probe design, yet its efficiency and stability need further verifications in NHPs. the mAGNET also provides an alternative for researchers who do not need to label fine cell subtypes while requiring safety. Scientists can pick the appropriate genetic targeting strategy according to their research needs.

## Viral Tools for Neuroscience Studies in Monkeys

The application of engineered viral tools in neuroscience started a new era of exploring neural circuits at the molecular, single synapse, and neuron populations levels. As the mainstay of modern neuroscience tools, viral vectors have the advantage of rapid and high-level exogenous gene expression, cell tropism, and propagation directionality that can be harnessed for neuronal tracing, manipulation, and monitoring.

**Fig. 2:**
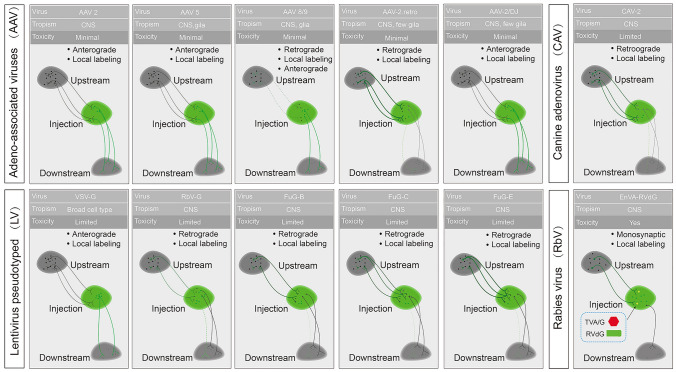
Key properties of representative viral tools for neuroscience studies in primates. Green cells and lines represent cells and fibers infected by viral vectors, and yellow dots indicate the co-expression of TVA/G and RVdG. The number of dots is proportional to the strength of local infection, the number and thickness of lines are proportional to retro/anterograde traffic strength, and dotted lines indicate possible projected performances. Retrograde, viral particles are usually taken up by axon terminals and retro-transported to the presynaptic soma; Anterograde, viral particles are usually taken up by cell bodies and transported to axon terminals; VSV-G, VSV glycoprotein, protein G; RbV-G, RbV glycoprotein, protein G; FUG-B, fusion glycoprotein type B; FUG-C, fusion glycoprotein type C; FUG-E, fusion glycoprotein type E; EnVA-RVdG, avian ASLV type A envelope protein (cognate ligand for TVA)- glycoprotein (G)-deleted rabies virus.

Viral tools have been widely applied in rodents, However, achieving viral-mediated robust, local, and pathway-selective exogenous gene expression in NHPs remains a challenging task, due to low expression levels, aggressive immune responses, and weak anterograde and retrograde transport efficiency. Here, we summarize recent progress in the use of viral tools for studies of primate neural circuits (Fig. 2).

### Adeno-associated Virus (AAV)

Recombinant adeno-associated viruses (AAVs), single-stranded DNA parvovirus with a payload capacity of approximately 3.5-5 kb, are key viral tools for neuroscience research. AAVs have a number of advantages, such as minimal toxicity, low immunogenicity, and long-term stable expression across multiple species. Newly developed AAV variants provide better results in terms of precise targeting; they are minimally invasive and can maximize cargo transgene capacity [36]. Different AAV serotypes have unique tropisms and infection properties, including organs tropism, cell types tropism, and direction of spread in neurons [37]. Here, we discuss the tropisms and biodistribution of known AAV capsid variants in primates as a reference for AAV serotype selection.

A systematic evaluation of AAV serotypes in the primate brain has indicated that AAV2 has the lowest transfection efficiency compared with other serotypes (AAV1, AAV5, AAV8 and AAV9) in the macaque cortex [11], AAV5 is the most effective vector in transducing cells and axonal terminals in the substantia nigra pars compacta (SNc) and striatum, followed by AAV1 and AAV2, while the least effective is AAV3 [38]. In this study, AAV2 has shown greater tropism to neurons (98%), while AAV5 transduces neurons and glial cells with equal efficacy. AAV8 and AAV9 exhibit local axonal terminal transport and retrograde transport across primate species. Yet, retrograde efficiency depends on the injection site: for instance, retrograde efficiency in the lateral geniculate nucleus (LGN) is relatively low compared to the SNc [39]. In conclusion, the infection results depend on many complex factors. Due to the limited sample sizes and experience, it is not yet possible to conclude the transduction performance of different AAV serotypes. Most researchers would pick suitable AAV serotypes based on their experience and personal preferences.

There are also many engineered variants derived from its parent capsids to suit customized study requirements. Among those capsid variants, AAV.DJ exhibits more robust and widespread local and axon terminal transport with neuronal tropism in the putamen and motor cortex of monkeys than AAV2 and AAV5 [40, 41]. AAV2.retro mediates stronger retrograde transport in the caudate and putamen of primates than AAV8 and AAV9, but in some brain regions, such as the occipital lobe and the cerebellum, it shows insufficient retrograde ability and robust axon terminal labeling [42]. AAV2.1 exhibits low neurotoxicity, and efficient gene transduction in large regions of the macaque brain [43]. AAV2.HBKO, AAV.CAP-B10, AAV.CAP-Mac, AAV.MaCPNS1, and AAV.MaCPNS2 have shown varying degrees of CNS neuronal tropism after intracranial injections in both marmosets and macaques [44, 46, 51].

In summary, AAVs are prominent gene delivery vehicles and have the potential to be tools for research on complex neural circuits in NHPs. Two major limitations of off-the-shelf AAV capsids would need to be addressed, both of which have been addressed in rodents: firstly, the efficiency of anterograde or retrograde circuit tracing in primates; secondly, the ability to cross the blood-brain barrier (BBB) and target brain cells of newborn or adult primates by intravenous administration. In rodents. AAV engineering can be done in three main ways: discovering naturally occurring AAV, rational design, and directed evolution [47, 53]. Among these, multiplexed-Cre recombination–based AAV targeted evolution (M-CREATE) has become the mainstream approach for capsid evolution, due to the high signal-to-noise ratio, mechanistic diversity, and screening capacity with cell type specificity. All reports published so far have applied the engineering strategy of screening in mice and validation in primates. Given the differences among the species considered, this approach is likely to cause early exits of ideal variants during the early screening rounds and lead to unpredictable infection patterns in NHPs. For instance, AAV.PHP.eB shows enhanced neuron-biased transduction after intracranial injections in C57BL/6J mice, but the BBB-penetrant ability can’t be kept in macaques, partly due to the absence of *Ly6a* in primates [45, 48-50]. In addition, AAV.MaCPNS1 and AAV.MaCPNS2 are transduced in the PNS in rodents, and both the PNS and CNS in NHPs [51]. Yet it is difficult to carry out directed evolution-based screening in primates due to a number of limitations. A combination of rational design and library screening may therefore be a better capsid modification and engineering approach in monkeys. It is worth noting that AAV.CPP.16, a recently reported rational design-based AAV9 variant by inserting cell-penetrating peptides (CPPs) between amino acids 588 and 589, has exhibited enhanced transduction efficiency after systemic delivery in young and adult macaques [52]. The reality, however, is that a rational design-based engineering strategy is hard to use as the molecular mechanisms of AAV tropism are still unclear [53]. More efforts in exploring the biological mechanisms of AAV infection are required to develop the next generation of AAV variant screening approaches in NHPs.

### Lentivirus Pseudotype (LV)

Lentivirus pseudotype (LVs) is a single-stranded RNA retrovirus with a capsid derived from HIV-1 and envelope proteins exchanged with those of another virus; in this way, the original viral characteristics are modified, affecting its tropism and transport direction [37]. There are two major types of lentivirus pseudotypes: VSV-G, in which the glycoprotein is the one from the vesicular stomatitis virus, and RbV-G, in which the glycoprotein is derived from the rabies virus. RbV-G has higher neurotoxicity than VSV-G. It is worth noting that RbV has outstanding retrograde traffic ability [54]. VSV-G has broad tropism and exhibits powerful local transduction in the primate CNS. NHP researchers have successfully transfected and manipulated the cortex, putamen, thalamus, and external globus pallidus (GPe) of monkeys *via* VSV-G [55-57]. Recently, new LV pseudotypes, HIV-1 lentivector fused with glycoprotein type B (FuG-B), glycoprotein type C (FuG-C), and glycoprotein type E (FuG-E), have started to achieve more efficient retrograde traffic and neuronal targeting [58]. Due to their excellent performance, these lentivirus pseudotypes have been selected for pathway-selective manipulation and connectome mapping in NHPs [41, 59, 60].

### Canine Adenoviruses 2 (CAV-2)

Canine adenoviruses-2 (CAV-2) are double-stranded DNA adenoviruses with a payload capacity of approximately 30 kb. In comparison to naturally-occurring AAV serotypes, CAV is taken up preferentially by terminals and then retrogradely transported from dendrites to soma [61]. Engineered CAV-2 has largely reduced neurotoxicity thanks to the removal of replication-related viral genes, which enables long-term "exogenous cargo" loading in a pathway-selective manner [37]. CAV-2 has been used to investigate neural circuits in primates. Bohlen *et al.* have shown that CAV-2 can mediate retrograde transgene expression of fluorescent proteins in motoneurons following craniofacial intramuscular injections in rhesus monkeys [62]. A subsequent study showed that CAV-2 is promising for circuit-specific [e.g., primary visual cortex (V1) to secondary visual cortex (V2)] delivery of Cre recombinase in primates [63]. CAV-2 provides another option to target and investigate specific cell types, but more research is required in order to optimize the performance of its transduction system.

### Rabies Virus (RbV)

Wild-type RbV, a negative-sense, single-stranded RNA rhabdovirus, can be trafficked in a retrograde manner across polysynaptic connections. The advent of the glycoprotein (G)-deleted rabies virus (RVdG) pseudotype, in which the propagation-related sequence “G” is replaced with a required sequence, such as GFP, led to the onset of a new era in monosynaptic input connectome research in rodents [64, 65]. RVdG has also been used in monkeys to depict the morphologies and connectomes of neurons in V1 [66]. Currently, the most widely-used RVdG is the avian ASLV type A envelope protein (EnvA)-pseudotype RVdG system, which can target specific cell types *via* TVA-EnvA recognition and monosynaptic retrograde transduction. In this system, the helper virus incorporates TVA and G, and EnvA-RVdG is injected into the desired region sequentially. RVdG particles only enter cells that co-express TVA and G (“starter cells”), then they replicate and propagate in a transsynaptic fashion by trans-complementation with G. Using the RVdG-mediated monosynaptic tracing system, researchers have mapped the direct monosynaptic inputs of V1 neurons and demonstrated the existence of brain regions and functional-specific feedforward to feedback (FF→FB) loops in the primate cortex [63]. Yet, RVdG presents several limitations in terms of labeling efficiency of presynaptic neurons and of potential cell toxicity, especially in higher mammals. The RVdG system needs to be engineered to achieve high efficiency and safety in order to study monosynaptic input connectomes in NHPs.

## Genetic Tools Used for Neuronal Activity Manipulation

In order to understand the assembly and inner workings of the mammalian brain, we need to decode complex dynamic brain networks by manipulating neural circuits artificially under normal and pathological conditions. The recent continuous development of genetic modulators has triggered a growth spurt of neural circuit research in small animals. Despite falling behind against rodents, some progress has also been made in primate optogenetics and chemogenetics. Here, we summarize the genetic manipulation tools that have been used in monkeys (Table 1).

**Table 1:** Overview of publications using genetic tools for manipulating neuronal activity in NHPs.

Methods	Viral vector	Exogenous gene	Promoter	Brain region	Date
Optogenetic activation	VSV-G	ChR2	α-CaMKII	Frontal cortex	2009
	AAV5, lentivirus	ChR2, SFO	hSyn, hThy1	M1, somatosensory	2011
	AAV5, lentivirus	ChR2, SFO	hSyn, hThy1	Parietal cortex	2011
	VSV-G	ChR2	EF1-α	Putamen, GPe, thalamus	2012
	AAV1	ChR2	SYN1	V1	2012
	Lentivirus	ChR2	CMV	Thalamus	2012
	AAV5	ChR2(H134R)	CAG, hSyn	FEF	2013
	AAV5	C1V1	α-CaMKII	PMC, FEF	2013
	AAV5, lentivirus	C1V1, ChR2	α-CaMKII	V1	2013
	AAV5	C1V1	α-CaMKII	LIP	2014
	AAV2	ChR2	CMV	FEF-SC	2015
	AAV5	C1V1	α-CaMKII	V1	2015
	AAV5	ChR2	α-CaMKII	PMv, M1	2015
	AAV9	ChR2	TH	Midbrain	2016
	AAV5	ChR2	α-CaMKII	LGN-V1	2016
	AAV6	C1V1, ChR2	α-CaMKII	PMC, M1	2016
	AAV5	ChR2	α-CaMKII	Perirhinal cortex	2017
	AAV	ChR2	L7	Cerebellum	2017
	AAV9, AAV1	ChR2	m*Dlx5/6* enhancer	V1	2020
	AAV2	ChrimsonR6	CAG	RGC	2020
	AAV.DJ	ChR2	CAG	M1	2020
	AAV5	ChR2, C1V1	α-CaMKII	FEF, PMd	2020
	AAV2	ChR2	CMV	CDt-GPe/SNr	2020
	AAV9	ChR2	hSyn	V1	2021
Optogenetic Inactivation	VSV-G	ArchT	CaMKII	Par, V1 cortex	2011
	AAV	ArchT	CAG	SC	2012
	AAV5	ArchT, eNpHR3.0	CAG, hSyn	FEF	2013
	AAV5, lentivirus	ArchT	α-CaMKII	V1	2013
	AAV8	ArchT	CAG	IT cortex	2015
	AAV8	Jaws	hSyn	FEF	2016
	AAV8	Jaws	α-CaMKII	MT	2018
	AAV5	eNpHR3.0	α-CaMKII	FEF	2020
Chemogenetic	Lentivirus	hM4Di	hSyn	OFC-Rh	2016
	AAV5	hM4Di	hSyn	Amygdala	2016
	Lentivirus, AAV2	hM4Di	hSyn, CAG	rmCD	2016
	AAV1	hM4Di	hSyn	PFC-MDI, Cd	2021
	AAV1, AAV2	hM4Di	CMV, hSyn	PFC, Putamen principal sulcus	2020
	Lentivirus	hM4Di	CMV	LC	2022
	AAV2	hM3Dq	CMV	Amygdala	2020
	AAV1	hM3Dq	TH	SN	2021
	AAV8	hM3Dq	α-CaMKII	POA	2023
Neurotoxin	AAV2, FuG-D	eTeNT	CMV, TRE	Spinal cord	2017
	AAV2, FuG-E	eTeNT	CMV, TRE	VTA-NAc	2020

### Primate Optogenetics

Optogenetics is a revolutionary neuroscience approach, which realizes temporally precise control of neural activity by genetically overexpressing opsins in neurons [67]. Since the discovery of the first light-gated protein, the halorhodopsin family (NpHR) in 1977 and channelrhodopsin-2 (ChR2) in 2002 [68], a large arsenal of naturally-occurring and synthetic opsins that enable optical control of neuronal activity with millisecond precision has been engineered.

Three main light-activated opsin types are used in monkeys: ChR2, a classic blue light-gated (470 nm) opsin with fast kinetics derived from *Chlamydomonas reinhardtii*; C1V1, a red-shifted opsin with enhanced photocurrent and fast kinetics; and step-function opsins (SFO), a variant of hChR2 (C128S) that switches on and off in response respectively to 470 nm and 542 nm light. The most widely used optical depolarizing opsins in monkeys are ChR2 and its enhanced variant ChR2 (H134R). Given that red light penetrates deeper into tissues than other visible wavelengths, C1V1 has also been chosen several times for optical stimulation in monkeys [69-71]. SFO and red-shifted CnChR1 (ChrimsonR) have also been selected for a few studies [13, 72]. More sensitive opsins with fast kinetics have been applied in mice with good results (e.g., Chrger2.0), and are expected to be useful in future monkey studies [73, 74].

Three main light-inhibitory types of opsins are used in monkeys: ArchT (archaerhodopsin-3), an outward proton pump that responds to yellow light (mostly 450-550 nm) [75]; eNpHR3, a yellow-light-sensitive chloride pump engineered from NpHR; and Jaws, a red-shifted opsin from *Halobacterium salinarum*, that enables robust and deeper neuronal silencing in red light (635 nm) [73, 76]. Jaws currently appears to be the most effective neuronal silencer for NHPs.

Han *et al.* first used ChR2 in 2009 to activate excitatory neurons in the frontal cortex of macaques, proving the feasibility of applying optogenetics to primates [55], and they transduced ArchT to neuronal membranes and axons in the visual cortex again in 2011, which resulted in the rapid and complete silencing of recorded neurons [56]. Similar opsin-mediated neuron depolarization/hyperpolarization has been further confirmed in the motor cortex, the somatosensory cortex, the parietal cortex, the visual cortex, the frontal eye field (FEF), the superior colliculus (SC), and in thalamic subnuclei [13, 57, 75, 77]. At this stage, the optogenetic perturbations are not yet able to induce any significant behavior. The work of Ohayon *et al*. has ruled out the hypothesis that optical stimulation in monkeys is subtle and subthreshold [78].

**Fig. 3.**
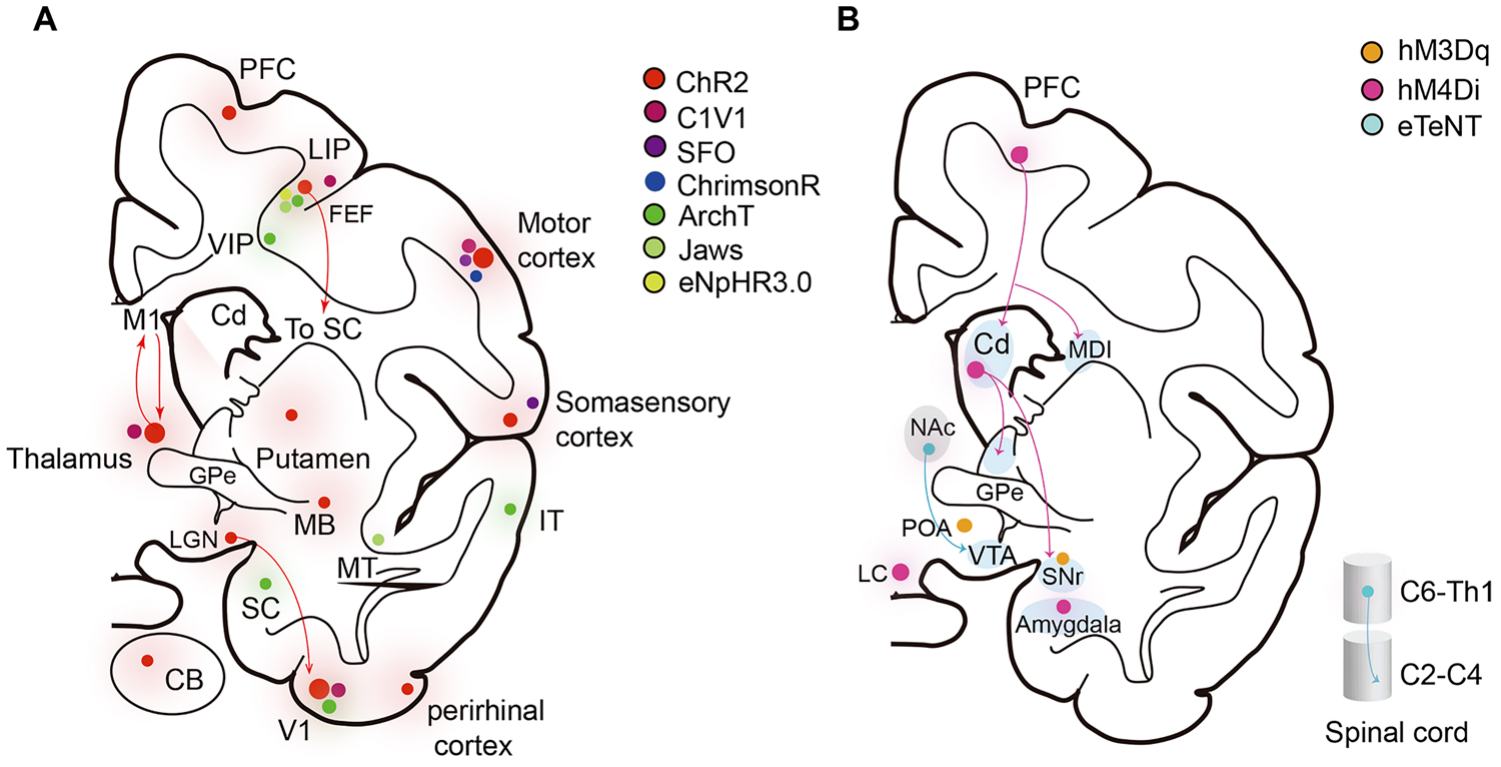
Application summary of genetic tools used for neuron manipulation in primates. **A** Opsins used for primate optogenetics. **B** Genetic tools used for primate chemogenetics and neurotoxins. The size of the dots is proportional to the injection frequency in the brain region for optogenetics and chemogenetics; the PFC, motor cortex, V1, and thalamus are popular regions. ChR2, channelrhodopsin-2; C1V1, red-shifted chimera; SFO, step-function opsins; ChrimsonR, red-shifted CnChR1; ArchT, archaerhodopsin-3; eNpHR3.0, halorhodopsin-kir2.1; Jaws, red-shifted cruxhalordoposin; eTeNT, tetanus neurotoxin; hM4Di, human Gi-coupled M4 muscarinic receptor; hM3Dq, human Gi-coupled M3 muscarinic receptor; PFC, prefrontal cortex; LIP, lateral intraparietal; FEF, frontal eye field; VIP, ventral intraparietal area; Cd, caudate nucleus; SC, superior colliculus; M1, primary motor cortex; GPe, globus pallidus, external segment; LGN, lateral geniculate nucleus; MT, middle temporal area; IT, inferior temporal cortex; V1, primary visual cortex; CB, cerebellum; MB, midbrain; NAc, nucleus accumbens, core; VTA, ventral tegmental area; SNr, substantia nigra pars reticulate; MDL, lateral mediodorsal thalamus; LC, locus coeruleus; POA, preoptic area.

Researchers have faced enormous challenges due to many obstacles, including limited transfection efficiency, inefficient light delivery and readout equipment, severe immune responses, and deficient injection protocols. After continuous improvement, NHP optogenetics has been transformed, fueled by developments in viral transudation efficiency (e.g., AAV2.1), surgical procedures (e.g., the convection-enhanced delivery (CED) method and window implantation), light delivery devices (e.g., the coaxial optrode), and readout approaches (e.g., fMRI and Ca^2+^ imaging) [43, 70, 79-83]. In the last decade, primates optogenetics have started an era of mesoscopic neuronal circuit neuroanatomy and functional studies with cell-type specificity (e.g., dopamine neurons and Purkinje cells) and pathway selectivity (e.g., FEF to SC and LGN to V1). Such studies have focused on face gender discrimination, direction discrimination, decision confidence, value-based learning, working memory, reward prediction, visual perception, saccade movement, and forelimb movements. Most of those studies have been completed in the primary visual system and the frontal system [15, 16, 27, 40, 57, 68, 69, 71, 72, 75-77, 83-97] (Fig. 3 A).

Today, primate optogenetics is undergoing a phase of rapid development, while still facing many challenges. The stability and efficiency of genetic modulators delivery largely depend on viral vectors. Engineering more useful viral vectors to ensure intense and stable expression of opsins is key to the modulation of neuronal activity. In addition, cell-type-specific manipulation would be a significant step toward decoding complex brain dynamics. The screening of specific *cis*-regulatory elements can lead us to achieve this goal [25, 26]. Developed opsins are also urgently needed to upgrade primate optogenetics; for instance, ChRs with enhanced light sensitivity 2.0 (Chrger2.0), a newly engineered opsin, enable neuron activation in mice without fiber-optic implantation, extending application directions for optogenetics experiments [74]. We believe that its use in monkeys would greatly reduce heat absorption in the brain and engage more neurons at lower power to induce behaviors.

### Primate Chemogenetics

Chemogenetics is another neuronal manipulation approach, in which engineered exogenous receptors expressed in target cells interact with exogenous ligands *via* the system or local drug administration [98]. The DREADDs used in chemogenetics are almost exclusively the human Gi-coupled M4 muscarinic receptor (hM4Di, inhibitory DREADDs) and human Gi-coupled M3 muscarinic receptor (hM3Dq, excitatory DREADDs), which are activated by clozapine-N-oxide (CNO), compound 21, and perlapine [98]. Once activated, hM4Di and hM3Dq attenuate or enhance neuronal excitation by binding to Gq/s or Gi proteins [99]. Compared to optogenetics, the development of primate chemogenetics is relatively lagging. The biggest disadvantage of chemogenetics is the inferior temporal resolution, which ranges from minutes to hours and does not allow investigation of the rapid dynamics of brain operations. In addition, chemogenetics has a relatively low spatial resolution. Despite the poor temporal resolution, chemogenetics is more powerful in driving robust behaviors (even motor behavior) than optogenetics. As such, DREADD technology is sometimes more desirable for reversible and large-scale control of cell populations, while being less invasive than optogenetics [99, 100].

Chemogenetics studies on NHPs are becoming increasingly popular. In these studies, hM4Di has been used to selectively block the motor cortex-propriospinal neurons (PNs) pathway and affect hand dexterity [100]; to disrupt the connection between the lateral prefrontal cortex (lPFC) and the striatum involved in inhibitory control [101]; to silence the dorsolateral prefrontal cortex (dlPFC)-lateral mediodorsal thalamus (MDL) or dlPFC-dorsal caudate nucleus (dCD) pathways involved in decision-making and working memory [102]; to inactivate the rostromedial caudate (rmCD) and PFC through the positron emission tomography (PET)-guided systemic administration of CNO [103, 104]. It has also been used to silence the PFC and induce spatial working memory deficits [105, 106]; and to reversibly inactivate the locus coeruleus (LC) and affect cognitive functions [107]. hM4Di, equipped with Cre-dependent viral tools, provides a powerful pharmacogenetic tool to selectively inactivate neuronal circuits. Oguchi *et al*. have used AAV5, which carries the "Cre-On" FLEX double floxed sequence, transferred hM4Di to the PFC, and then injected a retrograde vector that incorporated Cre-recombinase into the caudate nucleus (Cd). In this study, researchers have successfully suppressed specific neural circuits on a time scale ranging from minutes to weeks [101] (Fig. 3B). In a more recent study, the high-affinity and selective agonist deschloroclozapine (DCZ) has been reported for DREADD activation. This agonist displays high selectivity and fast kinetics in mice and monkeys [105]. There are also a few reports about chemogenetic activation using hM3Dq. Mimura *et al*. have used AAV1-encoded hM3Dq to activate TH^+^ neurons of the SNc in marmosets and observed rotation behaviors in a contralateral direction relative to the activated side [108]. Another study used AAV8-encoded HM3Dq to activate a group of neurons in the preoptic area (POA) and successfully drive hypothermia and cold defense in macaques [109] (Fig. 3 B). Overall, chemogenetic technologies are very promising, but there is still considerable room for improvement in terms of safety, off-target effects, efficiency, and specificity. For example, DREADD actuators have different potential effects on animals. One study showed that the monkey's working memory is impaired after olanzapine and DCZ injections [110]. Another example is that the subcellular localization of DREADDs depends on the DREADD/tag combination (e.g., the hM4Di‐mCherry is mostly expressed in the intracellular space, and the hM4Di‐haemagglutinin (HA) tag is mostly confined to the plasma membrane) [111]. A systematic parameter test before chemogenetic experiments is recommended.

### Neurotoxins

Neurotoxins are substances that can modulate the electrical activity and neurotransmitter release of neurons and destroy the nervous system by means of binding to specific membrane acceptors. Common examples of neurotoxin tools include nitric oxide (NO), botulinum neurotoxins (BoNT), and tetanus toxin (TeNT) [112]. Since most neurotoxins have a high affinity for specific molecular targets, they have long been used to study the anatomy, physiology, function, and disorders of the brain. And yet, the application of neurotoxins has some limitations, including slow inactivation time, off-target activity, the difficulty of extraction, and unclear effective concentration [113]. To date, two studies have reported a tetracycline-dependent enhanced tetanus toxin (eTeNT) system to realize selective and reversible lesions in the spinal cord and ventral tegmental area (VTA) respectively in NHPs. In this system, the virus that incorporates the Tet-on sequence is injected into the target brain region, while another retrograde virus, carrying the eTeNT sequence driven by the tetracycline (TRE) promoter, is injected into the downstream regions of interest. During the "doxycycline (Dox) on" period, Dox binds to the tetracycline transactivator (tTA) and activates the TRE promoter. The expression of eTeNT is then turned up in double-infected neurons that send signals to specific downstream regions. Then the connections between specific neural circuits are reversibly blocked in a Dox-dependent manner; these events do not occur in the absence of Dox [41, 59] (Fig. 3 B). Due to the natural deficits of neurotoxins, and the immaturity of the associated manipulation approach, their application for neural circuit studies in NHPs is relatively limited.

## Genetic Tools Used for Neuronal Activity Monitoring

The ability to monitor neuronal populations or single neurons with high spatiotemporal fidelity is crucial for a comprehensive understanding of the mammalian brain. Traditional approaches for neuron activity recording in primates are electrophysiology, fMRI, voltage-sensitive dye imaging (VSDI) and intrinsic optical imaging. Electrophysiology, a classic method in neuroscience, has high temporal fidelity but is limited by its low resolution regarding cell number, identity, and spatial localization. The oldest intrinsic optical imaging offers a minimally or non-invasive method to look inside the monkey brain, but its cell-type resolution, temporal fidelity, and signal-to-noise ratio are still lacking [114, 115]. VSDI can track neuronal activity with fast kinetics on the millisecond scale. However, it cannot be used for long-term recording due to high phototoxicity and low cell-type resolution due to the non-selective infection pattern of VSDI dyes [116–118].

The advent of genetically encoded optical indicators has enabled researchers to simultaneously record neurotransmitter concentrations, transmembrane voltage, the intracellular Ca^2+^ dynamics of specific neuron populations, and even fine structure. Novel genetically encoded optical tools have been used in small animals for years, but their application in primates lags behind. Recent advances in optical imaging in primates have shed new light on cortical information processing. Here, we summarize the genetically encoded tools used in primate neuronal circuit imaging to provide a reference for future research. Most of these tools are still at the proof-of-concept stage (Table 2).

**Table 2:** Overview of publications using genetic tools for monitoring neuronal activities of NHP.

Exogenous gene	Promoter	Brain region	Laboratory	Date
memTNXL	CD8	V1/V2	Ralph M. Siegel	2010
GCaMP6f	Thy1, TRE	The par cortex, S1/2	Tetsuo Yamamori	2015
GCaMP5G	hSyn	S1/2	Afonso C. Silva	2016
GCaMP6f	CaMKII, hSyn,CAG	V1	Boris V Zemelman	2016
GCaMP5G	hSyn	V1	Shiming Tang	2018
GCaNP6s	hSyn	V1	Shiming Tang	2018
GCaMP6f	Thy1, TRE	M1	Hideyuki Okano	2018
GCaMP5G	hSyn	V1	Shiming Tang	2018
GCaNP6s	hSyn	V1	Shiming Tang	2018
GCaMP5G	hSyn	V1	Tai Sing Lee	2018
GCaMP6f	h*DLX* enhancer	MT	Boris V. Zemelman	2019
iGluSnFR	hSyn	V1	Shiming Tang	2020
GCaMP5G	hSyn	V1	Cong Yu	2020
GCaMP6f	Thy1, TRE, CaMKIIα	PMd, M1	Jonathan J. Nassi	2020,2021
GCaMP6f	CaMKII, hSyn	PMd, M1	Krishna V.Shenoy	2021
GCaMP5G	hSyn	V4	Shiming Tang	2021
GCaNP6f	hSyn	V4	Shiming Tang	2021
GCaMP6s	hSyn	V1	Liping Wang	2022

### Genetically Encoded Calcium Indicators (GECIs)

Genetically Encoded Calcium Indicators (GECIs) have proven to be powerful imaging tools in NHPs, as they are capable of monitoring neurons via Ca^2+^ changes in neuron depolarization, during which the cytoplasmic Ca^2+^ concentration increases 10 or even 100 times. Two major types of GECIs have been used in monkeys: calmodium-based indicators, and troponinC-based indicators. memTNXL, a troponinC-based indicator, was used to monitor neurons as early as 2010 [119]. Yet memTNXL did not result in robust and fast fluorescence changes, and there have been no follow-up reports. Instead, improved versions of calmodium-based indicators have been applied in primates. The most widely used calmodium-based indicators in primates are GCaMP5G, GCaMP6s, and GCaMP6f [120-122]. Since the tTA-TRE inducible GCaMP6f expression system was first used for long-term imaging in anesthetized marmosets, several researchers have focused on this indicator [122]. GCaMP5G, GCaMP6s, and GCaMP6f, equipped with improved microscopes and window implantation, have been widely used to dissect neural circuits involved in social interactions, motor planning and execution, visual information processing, and sequential working memory [120-136]. Recent research has realized two-photon recording of interneurons in the marmoset cortex *via* the human *DLX5/6* enhancer-mediated GCaMP expression strategy. This approach, based on specific gene expression mediated by cis-regulatory elements, may be useful in revealing the functional characteristics of distinct neuron types [28].

In addition to the classical GCaMP version, a series of new Ca^2+^ indicators have been engineered to meet different experimental needs in rodents. For instance, jGCaMP7 improves single-spike detection; soma-targeted GCaMP by the endoplasmic reticulum and ribosome tethering reduces the level of crosstalk from the neuropil and improves imaging quality [137-139]. Newly engineered GECIs will provide novel and interesting insights for future primate studies.

### Genetically Encoded Transmitter Indicators (GETIs)

Genetically Encoded Transmitter Indicators (GETIs) can help us monitor the release of specific neurotransmitters and neuromodulators in the nervous system with high sensitivity and faster kinetics than Ca^2+^ signals [140]. A large arsenal of GETIs has been developed over the years. Among these, iGluSnFR is the most responsive sensor to a single action potential and dendritic boutons, with a high signal-to-noise ratio and temporal resolution when excited at 488 nm. Ju *et al.* have performed two-photon dendritic imaging with iGluSnFR in V1 of macaques and mapped a fine dendritic excitatory input atlas of neurons in the superficial layers of V1 [127]. The application of GETIs in primates is still in the early stage. Given the importance of neurotransmitters and neuromodulators in information processing, the development and application of GETIs for NHPs studies are crucial for research.

### Other Genetically Encoded Indicators

Genetically Encoded pH Indicators (GEPIs), such as super ecliptic pHluorin (SEP), can detect the release of synaptic vesicles mediated by pH changes between the vesicle lumen (pH 5.5) and the extracellular environment (pH 7.0) [140]. Genetically encoded voltage indicators (GEVIs) can also be used to investigate neuronal dynamics based on membrane potential changes in postsynaptic targets. Recent advances have led to the development of new voltage indicators with improved performance in voltage sensitivity, activation and inactivation speed, brightness, and cell toxicity. For example, mNeonGreen, a recently developed GEVI-fused proton-pumping rhodopsin, enables bright and fast neuron recording in mice and flies [141]. In addition, GEVIs can be coupled with other reporters, like opsin and neurotransmitter indicators, to enable all-optical interrogation of neuronal circuits [142]. These genetically encoded indicators have not yet been applied to the dissection of neural circuits in primates, and given their inherent properties, their application remains a challenge for studies in NHPs.

Nowadays, neural circuit functional research, either perturbing or observing, faces a fundamental and unavoidable dilemma. On one hand, even with the enhanced delivery efficiency of engineered AAVs, regulatory or recording units induced by local viral injection are still relatively small compared to the sizes of the brains of NHPs. On the other hand, multiple sophisticated neural circuits are mobilized systemically to guide behaviors in higher mammals. This makes it difficult to induce behaviors by relying solely on simple neuron manipulation as has been done in mice. Considering the particularity of monkey models, decoding and re-encoding information processing based on large numbers of cells would be perhaps more convincing. We recommend the further development of large optical control and imaging fields with high resolution and cell-type specificity in NHPs.

In order to address these limitations, we can improve the following aspects: (1) increased transfection range through surgical improvements and new technologies; (2) the development of BBB-penetrant AAVs to target brain cells in adult primates; and (3) the generation of transgenic optogenetic and calcium imaging monkey models. It is worth noting that transgenic monkey models can not only express the desired proteins in the whole brain, but their ability to indicate neuronal activity is also maintained over time by the constant synthesis of new proteins, meaning this method can support long-term studies for particular behaviors and diseases. To date, the first GCaMP marmoset study has been reported [143]. The use of optogenetics and GCaMP transgenic NHPs for functional neuroscience studies would be a milestone in this field.

Closed-loop investigation is another possible approach to quickly and intuitively build encoding and decoding models in primates. This concept has already been proven feasible in macaques [126]. Closed-loop interrogation of cortical neurons by optical activation and imaging still has a lot of room for technological improvement; for instance, improved opsins should be compatible with Ca^2+^ indicators, and they both should be expressed in the same cells.

## Conclusions

Genetic approaches offer powerful tools for studying the dynamics and connectivity of neural circuits in different cortical areas, and linking them to particular behaviors. The genome-editing technologies developed so far (e.g., the CRISPR/Cas system) have made it feasible to precisely generate gene-modified monkey models for neuroscience research [144]. In parallel, advances in single-cell transcriptomic and epi-genomic sequencing technologies have facilitated the understanding of gene expression regulatory networks and the cellular makeup of the primate brain, leading to the development of cell-type-specific tools in monkeys. Engineered viral capsids enable uniform and stable exogenous gene delivery. Neuronal activity modulators and indicators, driven by innovations in surgical procedures, light delivery devices, and readout equipment, are beginning to overcome past limitations and provide novel approaches to mesoscopic neural circuit research in primates.

Recently, the scientific community worldwide has undertaken the project of mapping the comprehensive brain cell atlas and mesoscopic connectivity atlas of primates [145, 146]. We believe that this project will help to develop improved genetic targeting tools and explore the neurobiological basis within neural circuits, including cellular components, wiring diagrams, and inner workings. In turn, efforts in the genetic functional analysis of neural circuits will facilitate a better understanding of the molecular-cellular and cell properties of the nervous system in NHPs, which are similar to those in humans. Progress in NHPs brain studies will enable people to better understand how the human brain works under normal physiological and pathological conditions.
